# Spontaneous remission of advanced progressive poorly differentiated non-small cell lung cancer: a case report and review of literature

**DOI:** 10.1186/s12890-019-0978-4

**Published:** 2019-11-11

**Authors:** Hee-Young Yoon, Heae Surng Park, Min Sun Cho, Sung Shin Shim, Yookyung Kim, Jin Hwa Lee

**Affiliations:** 1 Division of Pulmonary and Critical Care Medicine, Department of Internal Medicine, College of Medicine, Ewha Womans University, Seoul 25 Magokdong-ro 2-gil Gangseo-gu, Seoul, 07804 Republic of Korea; 2 Department of Pathology, College of Medicine, Ewha Womans University, 25 Magokdong-ro 2-gil Gangseo-gu, Seoul, 07804 Republic of Korea; 3 Department of Radiology, College of Medicine, Ewha Womans University, 25 Magokdong-ro 2-gil Gangseo-gu, Seoul, 07804 Republic of Korea

**Keywords:** Advanced stage, Chemotherapy, Prognosis, Immunity, Disease progression

## Abstract

**Background:**

Spontaneous remission (SR) of cancer is a very rare phenomenon of unknown mechanism. In particular, SR of non-small cell lung cancer (NSCLC) has been scarcely reported. We present the case of a 74-year-old woman with advanced, poorly differentiated NSCLC (highly expressing programmed death ligand-1 [PD-L1]) that progressed despite multiple lines of chemotherapy but then spontaneously remitted.

**CASE presentation:**

The patient presented with hemoptysis and was diagnosed with stage IIIA poorly differentiated NSCLC via bronchoscopic biopsy. She had an unremarkable medical history and moderate performance status. The initial treatment plan was surgery after neoadjuvant chemotherapy. Despite conventional chemotherapy, follow-up chest computed tomography (CT) showed gradual tumor progression and she decided against further treatment after fifth-line chemotherapy. However, the size of lung mass was markedly decreased on follow-up chest CT one year after ceasing chemotherapy. Also, follow-up positron emission tomography images showed decreased metabolic activity in the lung mass and a percutaneous biopsy specimen from the diminished lung mass revealed no viable tumor cells. A diagnosis of SR of NSCLC was confirmed, and the patient was without tumor progression on follow-up nine months later. Later, PD-L1 immunostaining revealed high positivity (> 99%) in initial tumor cells.

**Conclusion:**

Our case showing SR of poorly advanced NSCLC refractory to multiple lines of chemotherapy suggested the association between immunity and tumor regression.

## Background

Spontaneous remission (SR) of cancer is defined as partial or complete disappearance of a malignant tumor without any standard treatment or with inadequate treatment for cancer control. Cole and Everson first reported 47 cases of biopsy-confirmed SR of cancer, proposing the term and criteria for SR in 1956 [1]. Although neuroblastoma, bladder cancer, and lymphoma have shown relatively high incidences of SR [2], SR of non-small cell lung cancer (NSCLC) is rare, with only a few cases reported worldwide [3–19]. Here, we present a case of a 74-year-old woman diagnosed with advanced poorly differentiated NSCLC that spontaneously remitted after failure of multiple courses of chemotherapy.

## Case report

**Fig. 1:**
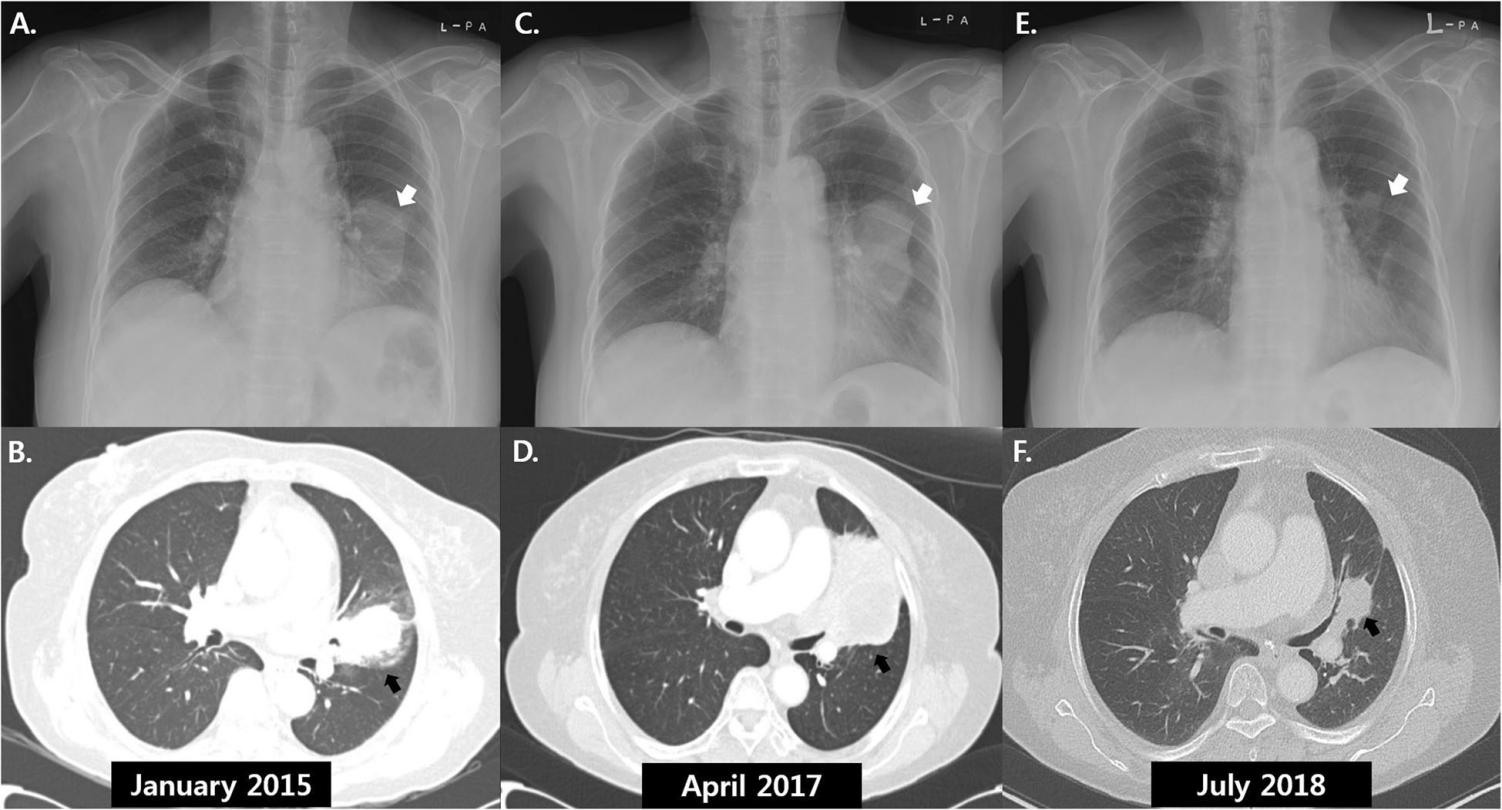
Serial chest radiographs and computed tomography scans of a 74-year-old woman with spontaneous remission of non-small cell lung cancer. **a**: Radiograph in January 2015 showed a left lung mass (white arrow). **b**: A 5.1 × 2.9 cm lung mass in the left upper lobe (black arrow) was observed on computed tomography (CT) images at the time of initial chest radiograph (**a**). **c** and **d**: After fifth-line chemotherapy, follow-up radiograph (**c**) and CT scans (**d**) in April 2014 showed an increase in size of the left lung mass. **e** and **f**: One year after the discontinuation of chemotherapy, significant reduction in size of the lung mass was demonstrated on follow-up radiograph (**e**) and CT scans (**f**) in July 2018

**Fig. 2:**
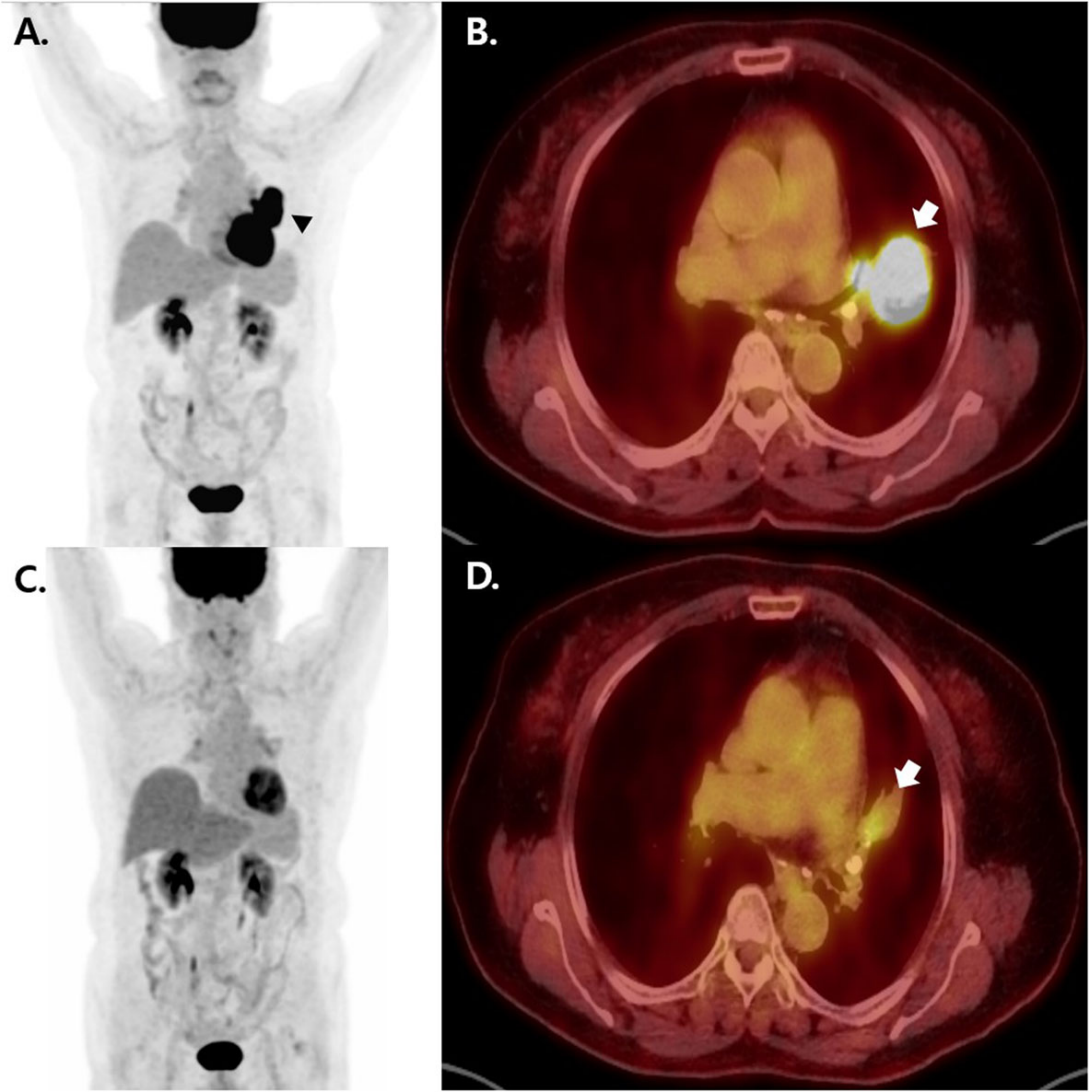
Comparison of in ^18^F-fluorodeoxyglucose positron emission tomography/computed tomography images before and after spontaneous remission of the left lung mass. **a** and **b**: A coronal positron emission tomography (PET) image in January 2015 revealed a mass with intense uptake (arrowhead) in the left lung (**a**) corresponding to a soft tissue density in a combined PET and CT fusion image (arrow). **c** and **d**: After the spontaneous remission, decreases in both size and uptake of the left lung mass were identified on a coronal PET image (**c**) and a combined PET and CT fusion image (arrow) (**d**) in October 2018

A 74-year-old woman was admitted to our institute with a history of a lung mass on a chest x-ray performed at another hospital for evaluation of recurrent hemoptysis. She had no relevant past medical or family history and was not taking any medication. She was a housewife and never smoked. She did not complain of respiratory symptoms except for hemoptysis. There was no history of occupational or environmental exposure. At the time of admission, her physical examination revealed decreased lung sounds in the left lower lung field. Laboratory testing revealed no abnormal results except for mild elevation of C-reactive protein (0.44 mg/dL, reference: 0–0.3 mg/dL)*.* Initial chest x-ray and computed tomography (CT) scan revealed a 5.1 × 2.9 cm lung mass in the left upper lobe with a surrounding halo of ground-glass attenuation (Fig. 1-a and b). There were also tiny lung nodules in the right upper and lower lobes that could not be ruled-out for malignancy and left hilar lymph node enlargement (11 L). Positron emission tomography/computed tomography (PET/CT) images showed a left lung mass with increased metabolic uptake (Figs. 2-a and b).

**Fig. 3:**
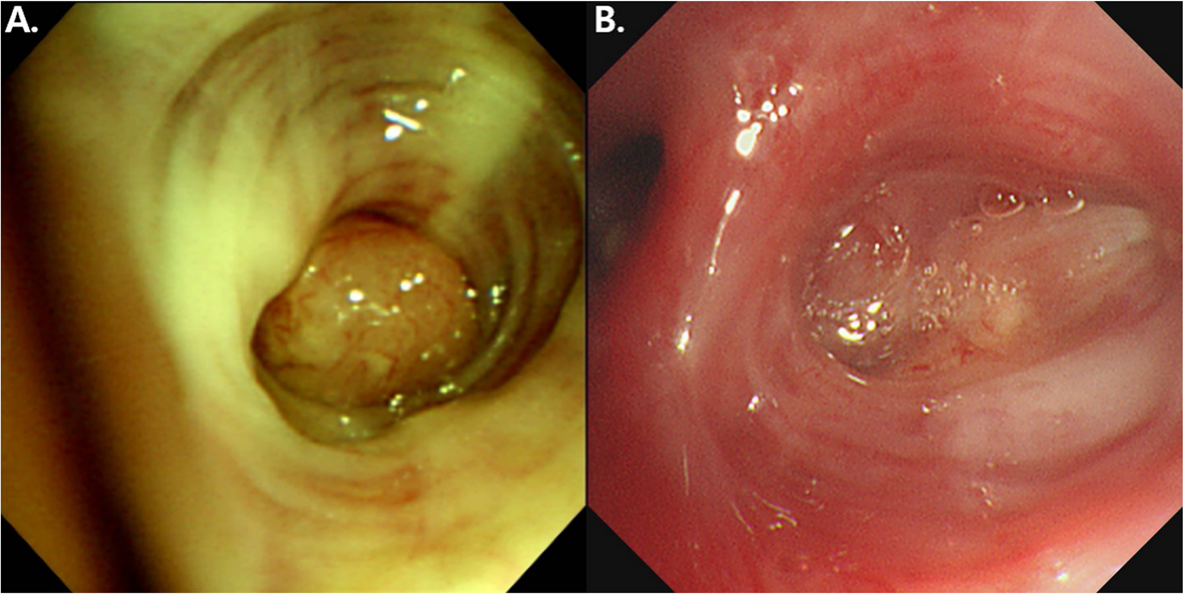
Comparison of bronchoscopic findings at the time of diagnosis of lung cancer and after spontaneous remission. **a**: On initial bronchoscopy, a polypoid endobronchial mass almost completely blocked the bronchial orifice of the lingular segment. **b**: After the spontaneous tumor remission, the entrance of the bronchus was replaced with a cicatricial lesion

**Fig. 4:**
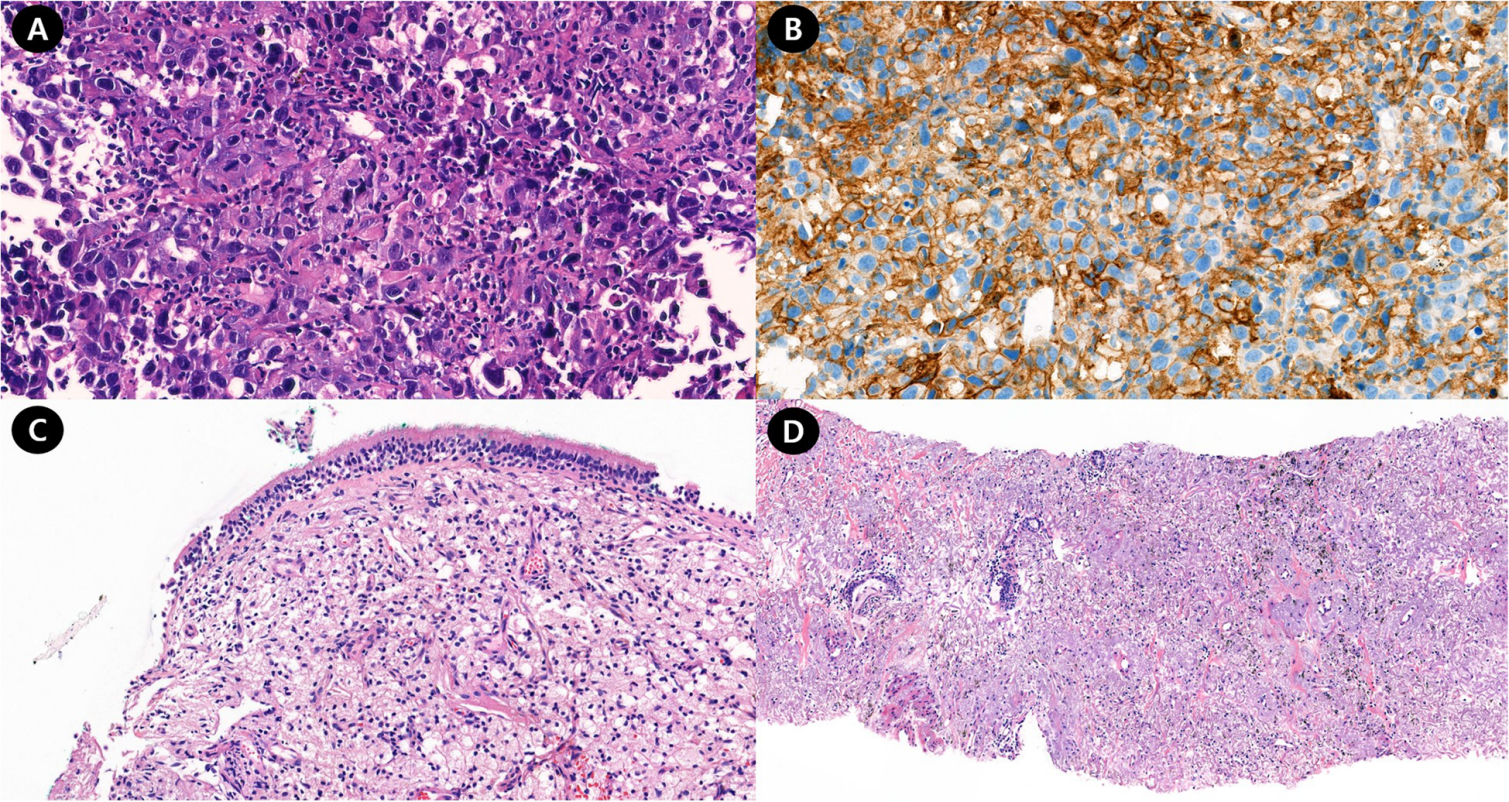
Histopathological findings of the initial and second bronchoscopic biopsies. **a**: The initial bronchoscopic biopsy showed poorly differentiated carcinoma with many tumor infiltrating lymphocytes. **b**: On immunohistochemical staining, nearly all the tumor cells showed PD-L1 expression. **c**: The second bronchoscopic biopsy showed chronic inflammation with foamy histiocytic infiltration, suggesting tumor regression. **d**: The percutaneous lung needle biopsy revealed marked fibroelastosis without tumor cells, another histologic feature indicating tumor regression

Diagnostic bronchoscopy revealed a polypoid mass with luminal obstruction of the left lingular inferior segmental bronchus (Fig. 3-a). Bronchoscopic biopsy of the lung mass was performed without complication. Histologic examination of an initial bronchoscopic biopsy showed poorly differentiated carcinoma with many tumor infiltrating lymphocytes (Fig. 4a). Immunohistochemical stains including cytokeratin (CK)-7, CK-20, thyroid transcription factor-1, napsin A, p63, p40 and CD56 were all negative. The initial pathological diagnosis was non-small cell carcinoma, not otherwise specified. The tumor was epidermal growth factor receptor wild type. An assay for anaplastic large-cell lymphoma kinase (ALK) rearrangement was not performed because the ALK inhibitor was not available. The patient was diagnosed with lung cancer in January 2015, long before the ALK inhibitor was approved for the treatment of lung cancer. Retrospective programmed death-ligand 1 (PD-L1) immunostaining (Ventana PD-L1 SP263 assay, Roche Diagnostics, Switzerland) showed high PD-L1 expression with a tumor proportion score of 99% (Fig. 4b).

After brain magnetic resonance imaging for cancer staging, which revealed a non-metastatic lesion, a tentative diagnosis of lung cancer with T3N1M0 (stage IIIA according to TNM seventh edition) was proposed. At first, the patient planned to undergo surgery after two cycles of paclitaxel plus carboplatin as neoadjuvant chemotherapy, inducing a decrease in the size of the lung mass from 5.1 × 2.9 cm to 4.1 × 1.5 cm. However, the patient developed drug-induced hepatitis with aspartate aminotransferase (AST) / alanine aminotransferase (ALT) of 110 / 182 IU/L due to a self-prescribed herbal medication, and the surgery schedule was delayed. For two weeks, waiting for the AST / ALT levels to drop, the lung mass slightly increased (4.1 × 2.6 cm), and she hesitated to undergo surgery. So afterwards she received additional two cycles of paclitaxel plus carboplatin, which means that she received a total of four cycles of paclitaxel plus carboplatin. However, the tumor still progressed, and fifth-line chemotherapy was sequentially administered until the fifth-line as follows: second-line for 4 cycles of gemcitabine plus carboplatin, third-line for 2 cycles of pemetrexed, fourth-line for 4 cycles of weekly docetaxel, fifth-line for 1 cycle of weekly vinorelbine. After a vinorelbine monotherapy, the patient refused further chemotherapy due to general weakness. At 4 months after discontinuation of chemotherapy, the size of the tumor on follow-up chest x-ray (Fig. 1-c) and CT (Fig. 1-d) was markedly increased (6.8 × 6.0 cm), directly invading left main pulmonary artery, left atrium, and left lower lobe. Thus, the patient again started irinotecan plus carboplatin as sixth-line chemotherapy. Although tumor size decreased after 4 cycles of chemotherapy, she decided to stop chemotherapy due to poor general condition and drug side effects and was scheduled for regular follow-up for tumor surveillance.

One year after discontinuation of treatment, a chest x-ray showed that the lung mass had decreased in size (Fig. 1-e). The patient had been taking herbal medication (Orostachys japonicus extracts) over the preceding few months. Chest CT taken during admission for disease status re-evaluation revealed the lung mass in the left lingular segment to have decreased in size to 3.6 × 2.5 cm (Fig. 1-f). PET/CT also demonstrated decreased size and metabolic activity of the lung mass (Figs. 2-c and d) and hilar lymph node. For further treatment planning, we performed bronchoscopy and intended repeat tumor biopsy, but only found fibrotic scar blocking the ligular segment instead of an endobronchial mass (Fig. 3-b). Histologic examination of the second bronchoscopic biopsy revealed chronic inflammation with foamy histiocytic infiltration (Fig. 4c). We subsequently conducted fluoroscopy-guided lung biopsy of the left lung mass. Percutaneous needle biopsy of the left lung lesion showed marked deposition of collagen and elastic fibers without tumor cells. (Fig. 4d). The histopathologic findings of second bronchoscopic biopsy and percutaneous lung needle biopsy were suggestive of tumor regression.

A diagnosis of SR of NSCLC was made, and the patient was without cancer progression on outpatient clinic follow-up at 9 months after the detection of SR.

## Discussion and conclusion

Our patient experienced SR of PD-L1-positive, poorly differentiated, advanced NSCLC refractory to conventional chemotherapy. SR of cancer is a unique phenomenon and few studies on SR are available. SR of cancer is estimated to occur in one in every 60,000 to 100,000 cancer patients according to the type of cancer [2, 20]. A review of the literature by Challis and Stam, including 741 SR cases between 1900 and 1987, documented that nine types of cancer (kidney, neuroblastoma, melanoma, choriocarcinoma, bladder, retinoblastoma, lymphoma, leukemia, and breast cancer) accounted for 69% of all SR cases, while SR of lung cancer only occurred in 2.6% of all patients with SR [20]. Because of the very rare occurrence of SR in lung cancer, only a few biopsy-confirmed NSCLC SR cases have been reported (Table 1).

**Table 1:** Literature review of spontaneous remission of histologically confirmed non-small cell lung cancer

Reference	Age/Sex	Cell type	Stage	Smoking	Comorbidities	Treatment
Present study	74/F	NSCLC, P/D	III	None	None	Herbal remedy after ceasing multiple line chemotherapy
Matsui et al. 2018 [11]*	56/F	SqCC, M/D	III	Smoker	CTD-ILD and autoimmune hepatitis	None
Ooi et al. 2018 [16]	77/M	NSCLC, P/D	III	None	none	None
Marques et al. 2017 [10]	75/M	AC, NA	I	Smoker	COPD, heart failure	None
Park et al. 2016 [17]	79/M	SqCC, NA	IV	NA	Hypertension, diabetes	None
Chung et al. 2015 [5]	67/M	SqCC, NA	IV	NA	None	Chemotherapy and herbal remedy
Ogawa et al. 2015 [15]	65/M	NSCLC, P/D	IV	Smoker	none	Radiotherapy
Menon et al. 2015 [12]	44/M	NSCLC, P/D	IV	Smoker	HIV	None except HAART on combined HIV infection
Lopez-Pastorini et al. 2015 [9]	76/M	LC, P/D	III	Smoker	hypertension	None
Hwang et al. 2013 [8]	62/M	NSCLC, P/D	III	Smoker	IPF, diabetes	None
Mizuno et al. 2011 [13]	62/M	LC, NA	IV	NA	None	None after surgery for initially stage I lung cancer
Furukawa et al. 2011 [6]	56/M	SqCC, NA	I	Smoker	COPD	None
Gladwish et al. 2010 [7]	81/F	SqCC, M/D	III	Smoker	Hypothyroidism	Herbal remedy (Essiac tea)
Nakamura et al. 2009 [14]	71/M	AC, P/D	III	NA	Anti-NY-ESO-1 immunity disease	None
Pujol et al. 2007 [18]	75/F	SqCC, NA	I	Smoker	Anti-Hu antibody syndrome, diabetes	None
Cafferata et al. 2004 [4]	68/M	AC, P/D	I	Smoker	COPD, ischemic heart disease	None
Kappauf et al. 1997 [3]	61/M	LC, P/D	IV	NA	None	None
Sperduto et al. 1988 [19]	61/M	SqCC, NA	IV	Smoker	COPD, basal cell cancer	None

*AC* Adenocarcinoma, *COPD* Chronic obstructive pulmonary disease, *CTD-ILD* connective tissue disease related interstitial lung disease, *F* Female; *HAART* Highly active antiretroviral therapy, *HIV* Human immunodeficiency virus, *IPF* Idiopathic pulmonary fibrosis, *LC* Large cell carcinoma, *M* Male, *M/D* Moderate differentiation, *NA* Not applicable, *SqCC* Squamous cell carcinoma, *P/D* Poor differentiation

The mechanism of SR remains unclear. Interestingly, poorly differentiated cancer is common among the reported cases of SR in patients with NSCLC. Light microscopy alone may be insufficient to diagnose poorly differentiated carcinoma, which has been reported to have a poor prognosis [21, 22]. The diagnosis of poorly differentiated carcinoma depends substantially on additional pathologist’s interpretation and adequate specimen size. The finding that many SR cases involved tumors showing poorly differentiated features despite testing with recently developed novel immunochemical markers suggests that the cancers of origin might have been misclassified. In addition, poorly differentiated NCLC has higher fluorodeoxyglucose (FDG) uptake at positron emission tomography and Ki-67 proliferation index compared with well-differentiated NSCLC [23]. Also, there have been several cases of poorly differentiated lung cancer accompanied by leukemoid reaction [24–26]. Inflammation is physiologically self-limiting; acute inflammation is terminated by activated neutrophils generating specialized pro-resolving mediators (SPM), including lipoxins, resolvins, protectins, and maresins, which are derived from essential fatty acids [27, 28]. There is in vitro evidence that SPM controls both innate and adaptive immunity by reducing the production of inflammatory cytokines (e.g., tumor necrosis factor-alpha and interferon-gamma) and memory B-cell antibody production [29, 30]. Considering the association between cancer progression and inflammation, increased cellular proliferation in poorly differentiated carcinoma might paradoxically induce the suppression of tumor growth via SPM, resulting in SR of cancer.

Unlike most other reported SR cases, which involved smokers, our patient was a non-smoker. Several studies recently reported superior efficacy of immunotherapy in smokers compared with non-smokers but did not clarify the mechanism of this effect [31]. Also, strong PD-L1 positivity and presence of tumor infiltrating lymphocytes (TIL), which are predictive biomarkers for immunotherapy [32], were identified in this case. Taken together, our findings and the results of previous studies support the association between immunity and cancer control.

Our patient was taking herbal medication (*Orostachys japonicus* extracts) after the discontinuation of chemotherapy. Gladwish et al. reported the case of a patient with SR of stable IIB NSCLC after receiving an herbal remedy (essiac tea) [7], which shows an antiproliferative effect on cancer cells at high concentration in vitro [33]. Chung et al. also reported the case of a patent with SR of NSCLC who took herbal medication during and after chemotherapy [5]. *Orostachys japonicus* is a flowering plant, containing several organic solvents including ethyl acetate with anti-cancer effects on human gastric cancer cells [34]. An in vivo model study also suggests the role of *Orostachys japonicus* in enhancing immunity by increasing immune cell propagation and production of immunity-related cytokines [35]. In our case, the tumor had many TIL and high PD-L1 expression, indicating that the patient had an antitumor immune response and had been eligible for treatment with an immune checkpoint inhibitor. Although no data are available to demonstrate the efficacy of *Orostachys japonicus* in humans, SR in our cases might be influenced by *Orostachys japonicus* intake.

Because our patient had received multiple cycles of chemotherapy before the occurrence of SR, there was a possibility of pseudo-progression or delayed response to chemotherapy. However, the chemotherapy regimen had changed several times, and tumor regression was observed one year after the last treatment, suggesting a high probability of SR. In addition, all of the chemotherapeutic agents administered to our patient would be less likely to show pseudo-progression because they are conventional drugs rather than immune checkpoint inhibitors.

In conclusion, we document a case of SR in a patient with advanced NSCLC refractory to conventional chemotherapy. Although the precise mechanism of SR in this case is unknown, the alteration of immunity might be an explanation. A single case cannot lead to a definite conclusion; nevertheless, our case indicates the importance of immunity in lung cancer control. Further well-designed animal model studies are needed to explore these findings.

## Data Availability

Any data generated and/or analysed during the current study are available from the corresponding author on reasonable request.

## Abbreviations

CK: Cytokeratin
CT: Computed tomography
FDG: Fluorodeoxyglucose
NSCLC: Non-small cell lung cancer
PD-L1: Programmed death ligand-1
PET/CT: Positron emission tomography/computed tomography
SR: Spontaneous remission
